# Effects of different amounts of okara on texture, digestive properties, and microstructure of noodles

**DOI:** 10.1002/fsn3.4007

**Published:** 2024-04-22

**Authors:** Zhongwen Cao, Lingchen Zhou, Sumin Gao, Cheng Yang, Xiangren Meng, Zhao Liu

**Affiliations:** ^1^ School of Tourism and Cuisine Yangzhou University Yangzhou China; ^2^ Key Laboratory of Chinese Cuisine Intangible Cultural Heritage Technology Inheritance Ministry of Culture and Tourism Yangzhou China; ^3^ School of Food and Engineering Yangzhou University Yangzhou China; ^4^ Engineering Technology Research Center of Yangzhou Prepared Cuisine Yangzhou China; ^5^ Fuzhou Polytechnic Fuzhou Polytechnic Fuzhou China

**Keywords:** digestion characteristics, fresh noodles, microstructural, soybean residue, textural properties

## Abstract

As a byproduct of manufacturing soybeans, okara is high in dietary fiber, protein, and fats, and it contains all of the essential amino acids. Wheat, the primary ingredient in noodles, will lose nutrients during manufacturing, creating an imbalance in nutrients. This experiment is for the purpose of studying the effects of okara on quality, antioxidant properties, amino acid content, resistant starch (RS) content, and microstructure of noodles. The results indicate that the addition of 9% okara noodles increased hardness and adhesiveness by 107.19% and 132.14%, respectively, and improved ABTS free radical scavenging activity by 60.78%. The addition of 12% okara noodles increased the DPPH free radical scavenging ability by 23.66%, reduced the rapidly digestible starch (RDS) content of the noodles to 21.21%, and the resistant starch content increased to 44.85% (*p* < .05). Therefore, to address the issue of nutritional imbalance in wheat noodles without compromising the quality of the noodles, it is recommended to add 9% or 12% okara for the preparation of nutritionally fortified noodles.

## Graphical abstract

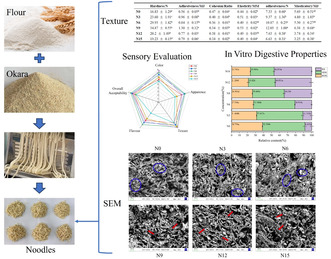

## 1 INTRODUCTION

Okara, also known as soybean dregs, is a byproduct obtained from *Glycine max* that is processed from soybeans. It consists of the insoluble portion leftover from soy milk or tofu production. It is inexpensive and easily obtainable (Vong & Liu, 2016). In Asia, there is a relatively high demand for soy milk and tofu, resulting in a high yield of okara. Approximately 1.2 kg of okara can be produced from every 1 kg of soybeans. China, Japan, and South Korea alone can produce 4 million tons of okara annually, making it a noteworthy byproduct in the food processing industry (Asghar et al., 2023).

Currently, most okara is disposed of as industrial waste, with only a small portion being used as animal feed. Okara has a rich array of nutritional components, containing 40%–65% dietary fiber, 25% protein, and 10% oil (dry basis). It also contains all essential amino acids, making them a good source of nutrition (Tang et al., 2021). In addition, okara can supply the limiting amino acids in cereal foods, especially lysine. As a result, okara has gained global attention due to its favorable chemical composition in recent years (Asghar et al., 2023; Pan et al., 2018). Additionally, studies have verified that okara's dietary fiber content possesses hypolipidemic, hypoglycemic, and antioxidant properties (Huang et al., 2022).

Noodles are a widely eaten staple food not only in Asia but even around the world (Han et al., 2020). In Asia, almost 40% of the wheat grown is consumed in the form of noodles (Du et al., 2021). However, when wheat is processed into noodles, nutrients are lost, resulting in nutritional imbalance (Parenti et al., 2020). Additionally, the main component of wheat products, starch, has been classified as rapidly digestible starch (RDS) that is digested within 20 min, leading to a relatively high blood sugar response compared to other grain products (Tang et al., 2021). To enhance the nutritional value and offer wheat flour noodles a unique flavor, a number of studies have looked into substituting black highland barley, Tartary buckwheat, and sorghum for wheat flour (Xie et al., 2023). As a result, substituting okara for wheat flour in common dishes not only increases the value of okara but also addresses the nutritional deficits of foods made from refined rice, resulting in the creation of a product with beneficial health effects.

## 2 MATERIALS AND METHODS

### 2.1 Materials and chemicals

Wheat flour with a crude protein content of 12.2% (N × 6.25) was purchased from Yihai Kerry Grain Industry Co., Ltd. (Shanghai, China). Fresh okara with a crude protein content of 19.35 ± 0.56% (N × 6.25) was obtained from the Beverage and Food Management Center at Yangzhou University (Yangzhou, China). The okara was dried in an oven (DHG‐9148A, Shanghai Jinghong Experimental Equipment Co., Ltd., China) at 60°C for 6 h. The dried okara was then ground using a grinder (H7d, Xiamen Hehui Electronic Technology Co., Fujian, China) and sieved through an 80‐mesh screen. 1,1‐Diphenyl‐2‐picrylhydrazyl, α‐amylase, and diammonium 2,2′‐azinobis (3‐ethylbenzothiazoline‐6‐sulfonate) were purchased from Shanghai Yuan Ye Biotechnology Co., Ltd. (Shanghai, China), and starch glucosidase was purchased from Shanghai Macklin Biochemical Technology Co., Ltd. (Shanghai, China). All chemicals used were of analytical grade.

#### 2.1.1 Preparation of okara noodles and samples

Based on the total amount of mixed powder (wheat flour and okara powder) of 100 g, 1 g salt, and 50 g water were added. The method of Zhang et al. (2022) was modified and combined with previous experiments on the noodle formula. Wheat flour was substituted with okara at six increasing concentrations (0%, 3%, 6%, 9%, 12%, 15%) to make mixed flour samples N0, N3, N6, N9, N12, and N15. The mixture powder was mixed with salt water, kneaded in a mixer (MK‐HKM200, Xiamen Jiansen Electric Co., LTD., China) for 5 min, then wrapped the dough in plastic wrap and left in an airtight container at 25°C for 20 min. After that, the dough was pressed with a dough press (BJM‐5, Deqing Baijie Electric Appliance Co., LTD., Zhejiang), and the roll gap of the dough machine was adjusted to 4.0, 3.0, 2.5, 2.0, 1.5, 1.0 mm in turn, and the surface was pressed 4 times for each roll gap. Finally, cut the pieces into 2.5 mm wide noodles. For the measurement of color difference, the surface is cut to 6 × 8 mm. The noodle samples were placed on a freeze dryer (Model 001‐10NA0024, Ningbo Xinzhi Equipment Shopping Technology Co., Ltd., China) after cooking and freeze‐dried for 48 h. The powdered samples were then kept at −80°C for future use after being freeze‐dried, including oxidation resistance, vitro digestive properties, amino acid content, and microscopic structure.

### 2.2 Color measurements of cooked noodles

The color difference in noodles was determined with the method of Wen et al. (2023), with minor changes. For the measurement of color difference, the first step is to cut the slices into 6 × 8 mm pieces. Then, the color of each group of fresh slice pieces is measured by a colorimeter (CR‐410, KONICA MINOLTA, INC. Japan), and the values of raw noodles are recorded as $L_{1}^{*}$, $a_{1}^{*}$, and $b_{1}^{*}$. The colorimeter was set to the illuminant C, observer angle of 2°, and aperture size of 50 mm after calibrating with a standard black and white plate. Finally, each group of slice pieces is cooked for the optimal cooking time (center core just disappeared), rinsed, and cooled, and the color is measured again to record the $L_{2}^{*}$, $a_{2}^{*}$, and $b_{2}^{*}$. The instrument is calibrated using a standard black and whiteboard. Each sample is tested three times for repeatability. The formula was used to calculate the whiteness index (WI):

(1) $$\mathrm{WI}=100-\sqrt{{\left[100-\left(L^{*}\right)\right]}^{2}+{\left(a^{*}\right)}^{2}+{\left(b^{*}\right)}^{2}}.$$

### 2.3 Texture profile analysis of cooked noodles

A texture analyzer (TPA) is used to examine the textural qualities of the noodles. The texture of the noodles is based on the method of Cao et al. (2017), with some adjustments. The texture analyzer (TMS‐Pro/touch, China) is used in conjunction with a P/35 cylindrical probe for the operation. After cooking each group of noodle samples at their respective optimal steaming time, they were rinsed with water for 10 s. The surface moisture of the noodles was then drained, and the noodles were placed parallel to the probe for testing. The testing was conducted at a speed of 1.0 mm/s before, during, and after deformation. The deformation of the noodles was set to 60%, with an automatic trigger type and a trigger force of 0.5 N. The time interval between two compressions was set to 1 s. Each group of noodle samples was cooked and tested separately, with at least 3 repetitions for each sample.

### 2.4 Sensory evaluation

The method of sensory evaluation is slightly modified by referring to the method of Ding et al. (2021). The noodle samples with the same length were selected and boiled in 100°C water to the optimal cooking time (center core just disappeared). The samples were dried for not more than 10 min in tightly covered plastic food containers before testing. Sample noodles were evaluated by 20 students and staff of the School of Tourism and Cuisine, Yangzhou University. All the samples were coded in a randomized order and evaluated by a 9‐point hedonic scale (1: dislike extremely, 5: neither like nor dislike, 9: like extremely). The different sensory attributes included color, appearance, taste, flavor, hardness, toughness, and overall acceptability.

### 2.5 Oxidation resistance

#### 2.5.1 DPPH radical scavenging activity

The DPPH free radical scavenging activity of the noodle samples was assessed by the method of Zhu et al. (2019) with minor modifications. A quantity of 1 g of freeze‐dried noodle sample was added to 20 mL of 80% methanol solution. A volume of 20 mL of 80% methanol solution was mixed with 1 g of freeze‐dried noodle sample. After ultrasonic extraction for 5 min (WH‐030S, Suzhou Chuanghui Electronics Co., Ltd., China), constant temperature shock at 60°C for 30 min (SHZ‐C, Shanghai Boxun Medical Biological Instruments Co., Ltd., China), centrifuged at 5000 r/min for 5 min, the supernatant was collected and set aside for further analysis.

To determine the DPPH scavenging ability of the 2 mL sample extract, it was mixed with a 2 mL 1,1‐diphenyl‐2‐picrylhydrazyl (DPPH) ethanol solution (71 μmol/L) and allowed to react for 30 min at room temperature in the absence of light. The sample's absorbance was then measured at 517 nm. The formula was used to calculate the DPPH scavenging ability:

(2) $$\text{DPPH radical scavenging activity}\;\left(\%\right)=\frac{A_{b}-A_{s}+A_{c}}{A_{b}}\times 100,$$

where: *A*_s_ is the absorbance value of the mixture of 2 mL sample extract and 2 mL DPPH ethanol solution. *A*_b_ is the absorbance value of the mixture of 2 mL anhydrous ethanol and 2 mL DPPH ethanol solution. *A*_c_ is the absorbance value of the mixture of 2 mL sample extract and 2 mL anhydrous ethanol.

#### 2.5.2 ABTS radical scavenging activity

For the measurement of the ABTS scavenging ability in the noodle sample, the method was determined according to Cai et al. (2022), with slight modifications. Diammonium 2,2′‐azinobis (3‐ethylbenzothiazoline‐6‐sulfonate) (ABTS) solution (7 mmol/L) was mixed in a 1:1 ratio with potassium persulfate solution (2.45 mmol/L) to make the ABTS solution. The mixture was thoroughly mixed and stored at 4°C in the dark for 12 h. In total, 3.9 mL of the ABTS solution was mixed with 0.1 mL of the extract to measure the ABTS scavenging activity. The mixture was allowed to react in the dark for 6 min before measuring the absorbance at 734 nm. The following is the formula for estimating ABTS scavenging activity:

(3) $$\text{ABTS radical scavenging activity}\;\left(\%\right)=\frac{A_{b}-A_{s}+A_{c}}{A_{b}}\times 100,$$

where *A*_s_: Absorbance value of the mixture of 0.1 mL sample extract and 3.9 mL ABTS solution. *A*_b_: Absorbance value of the mixture of 0.1 mL deionized water and 3.9 mL ABTS solution. *A*_c_: Absorbance value of the mixture of 2 mL sample extract and 3.9 mL deionized water.

### 2.6 In vitro digestive properties

The in vitro digestive properties of noodles were examined using the method of Li et al. (2022), with minor changes. Firstly, 500 mg of freeze‐dried cooked noodle powder was weighed and added to sodium acetate buffer solution (10 mL, 0.2 mol/L). After thorough mixing, the mixture was reacted in a boiling water bath for 30 min, followed by rapid cooling to room temperature. The mixture was then incubated for 5 min at 37°C. The combination was treated with α‐amylase (1 mL, 290 U/mL) and amyloglucosidase (4 mL, 15 U/mL). Subsequently, 0.5 mL aliquots of the mixture were taken at 0, 10, 20, 30, 60, 90, 120, and 180 min. Each aliquot was mixed with 4.5 mL anhydrous ethanol, and centrifuged at 4000 rpm for 15 min, and the supernatant was collected as the test solution. The supernatant glucose content was measured using the 3,5‐dinitro salicylic acid (DNS) technique. The following formulas were used to calculate the contents of RDS, SDS, and RS:

(4) $$\mathrm{SDS}\;\left(\%\right)=\frac{\left(G_{120}-G_{20}\right)\times 0.9}{\mathrm{TS}}\times 100$$

(5) $$\mathrm{RDS}\;\left(\%\right)=\frac{\left(G_{20}-\mathrm{FG}\right)\times 0.9}{\mathrm{TS}}\times 100$$

(6) $$\mathrm{RS}\;\left(\%\right)=\frac{\left[\mathrm{TS}-\left(\mathrm{RDS}+\mathrm{SDS}\right)\right]}{\mathrm{TS}}\times 100,$$

where G_20_ represents the glucose content generated after 20 min of enzymatic hydrolysis of the sample; G_120_ represents the glucose content generated after 120 min of enzymatic hydrolysis of the sample; FG represents the glucose content of the sample before enzymatic hydrolysis; and TS represents the total starch content.

### 2.7 Amino acid content

The determination of free amino acid content in noodles followed the method of Mudgal and Singh (2022), with slight modifications. To begin, accurately weigh 2 g of freeze‐dried noodle powder sample and add it to a 10 mL ethanol–water solution, followed by 30 min of ultrasonic shaking and 30 min of standing. The filtrate is centrifuged at 10,000 rpm/min for 10 min after passing through a double‐layer filter paper, and the supernatant is collected for later use. Finally, an Agilent liquid chromatograph (Ag1100) is used for measurement. The chromatographic conditions are as follows: the chromatographic column is ODS HYPERSIL column (250 mm × 4.6 mm), the column temperature is 40°C, the elution rate is 1.0 mL/min, and the ultraviolet detection wavelength is 262 nm.

### 2.8 Microstructural analysis

The observation of the microscopic structure of the noodles was conducted based on the method by Han et al. (2022), with slight modifications. Firstly, the cooked noodle samples were cut into 10 mm segments and placed in a 4% glutaric dialdehyde at 4°C for 12 h. Then, after dehydration treatment using ethanol gradient, CO_2_ critical point drying, and gold sputtering, and using a Zeiss GeminiSEM 300 field emission scanning electron microscope (from Carl Zeiss, Germany) to take photographs and observe under a magnification of 300×.

### 2.9 Statistical analysis

Each experiment was repeated three times. The data is presented as mean ± standard deviation. Significance difference analysis and correlation analysis were performed using IBM SPSS Statistics 26 (SPSS Inc., Chicago, IL, USA). Graphs were plotted using Origin 2022 (OriginLab, Hampton, MA, USA). Duncan's multiple comparison test was used to determine the statistical differences between the means (*p* < .05).

## 3 RESULTS AND DISCUSSION

### 3.1 Color measurements of raw and cooked noodles

Noodles are one of the most important staple foods for humans worldwide, and the color of noodles is a key determining factor for consumer acceptance (Morris, 2018). Another important comparison is the color variations of the noodles before and after cooking. The results are shown in Table 1. It can be seen that there is a significant difference between the control group noodles and the soybean bran noodles (*p* < .05). The brightness value ($L_{1}^{*}$) of raw noodles is higher than that of the control group, and the yellowness value ($b_{1}^{*}$) and redness value ($a_{1}^{*}$) of raw noodles also gradually increase with the increasing amount of soybean bran. This result may be due to the light yellow color of the dried soybean bran (Taruna & Astuti, 2018), resulting in a slight yellow color in the noodles after adding soybean bran. After cooking, the $L_{2}^{*}$ value of the noodles initially increases and then decreases, with the highest value observed in group N9, while both $b_{2}^{*}$ and $a_{2}^{*}$ show an upward trend. This may be due to the cooking process, which causes the noodles to heat up and absorb water, resulting in starch gelatinization and the more prominent color of the okara (Zhang et al., 2022). The whiteness index can be used to explain the overall color of the okara noodles. The whiteness index of the noodles dropped as the amount of soybean residue increased, reaching its lowest value at N15. Dried okara fiber has a pale yellow look and can retain its color even when combined with wheat flour. Consequently, this helps to explain why noodles with a higher okara concentration have a lower whiteness index.

**TABLE 1:** Color measurements of raw and cooked noodles samples with different amounts of added okara.

	Raw noodles				Cooked noodles			
	$L_{1}^{*}$	$a_{1}^{*}$	$b_{1}^{*}$	WI_1_	$L_{2}^{*}$	$a_{2}^{*}$	$b_{2}^{*}$	WI_2_
N0	79.49 ± 0.23^Ac^	−1.87 ± 0.02^Ae^	12.76 ± 0.21^Ae^	75.77 ± 0.3^Ab^	70.65 ± 0.72^Bd^	−2.08 ± 0.03^Be^	7.10 ± 0.67^Be^	69.73 ± 0.55^Bb^
N3	78.56 ± 0.55^Ad^	−1.65 ± 0.04^Ad^	14.44 ± 0.54^Bd^	74.09 ± 0.15^Acd^	74.30 ± 0.36^Bc^	−1.87 ± 0.07^Ad^	18.47 ± 0.74^Ad^	74.12 ± 0.77^Aa^
N6	84.13 ± 0.06^Ba^	−0.11 ± 0.05^Bc^	17.65 ± 0.07^Bc^	76.26 ± 0.04^Aa^	77.01 ± 0.32^Aab^	1.37 ± 0.15^Ac^	25.07 ± 0.23^Ab^	65.96 ± 0.16^Bd^
N9	82.85 ± 0.21^Ab^	0.27 ± 0.05^Bb^	19.73 ± 0.46^Ba^	73.85 ± 0.48^Ade^	77.32 ± 0.36^Ba^	1.36 ± 0.09^Ac^	24.25 ± 0.15^Ac^	66.77 ± 0.17^Bc^
N12	82.73 ± 0.11^Ab^	0.29 ± 0.01^Bb^	19.98 ± 0.02^Ba^	73.59 ± 0.08^Ae^	76.26 ± 0.29^Bb^	1.76 ± 0.11^Ab^	25.73 ± 0.27^Ab^	64.95 ± 0.21^Be^
N15	82.96 ± 0.05^Ab^	0.95 ± 0.01^Ba^	19.19 ± 0.06^Bb^	74.31 ± 0.02^Ac^	74.63 ± 0.46^Bc^	3.36 ± 0.05^Aa^	27.67 ± 0.32^Aa^	60.93 ± 0.44^Bf^

*Note*: Data were expressed as means ± standard deviations (*n* = 2). Different values of the small letters represent significant differences in the same column (*p* < .05) and the different large letters represent a significant difference between the raw and the cooked noodles (*p* < .05).

Abbreviations: N0, Wheat noodles; N3, Noodle with 3% okara; N6, Noodle with 6% okara; N9, Noodle with 9% okara; N12, Noodle with 12% okara; N15, Noodle with 15% okara; WI, Whiteness index.

### 3.2 Texture profile analysis of cooked noodles

The text in Table 2 shows the texture characteristics of noodles with added okara. The textural properties of the noodles are meanly influenced aside from the matrix structure network of starch, gluten, other proteins, fibers, and other additional components (Cao et al., 2021). The two most important textural characteristics that influence customers are elasticity and hardness (Wang et al., 2021). With increasing okara content, the hardness and adhesiveness of the noodles significantly increase (*p* < .05), while the cohesiveness and elasticity show no significant differences except in the control group (*p* > .05).

**TABLE 2:** Texture characteristics of cooked noodles samples with different amounts of added okara.

	Hardness/N	Adhesiveness/mJ	Cohesion/ratio	Elasticity/mm	Adhesiveness/N	Masticatory/mJ
N0	16.83 ± 1.29^e^	0.56 ± 0.05^d^	0.47 ± 0.04^a^	0.44 ± 0.02^b^	7.33 ± 0.60^c^	5.69 ± 0.51^ab^
N3	23.60 ± 1.91^c^	0.96 ± 0.08^b^	0.40 ± 0.04^b^	0.51 ± 0.03^a^	9.37 ± 1.36^b^	4.80 ± 1.03^b^
N6	29.93 ± 1.42^b^	0.84 ± 0.13^bc^	0.36 ± 0.01^b^	0.49 ± 0.02^ab^	10.87 ± 0.25^a^	5.30 ± 0.27^ab^
N9	34.87 ± 0.55^a^	1.30 ± 0.12^a^	0.34 ± 0.03^b^	0.53 ± 0.02^a^	12.03 ± 1.00^a^	6.38 ± 0.68^a^
N12	20.2 ± 1.49^d^	0.77 ± 0.03^c^	0.38 ± 0.01^b^	0.49 ± 0.03^ab^	7.63 ± 0.38^c^	3.74 ± 0.34^c^
N15	19.23 ± 0.15^d^	0.79 ± 0.06^c^	0.34 ± 0.02^b^	0.49 ± 0.04^a^	6.63 ± 0.31^c^	3.25 ± 0.38^c^

*Note*: Data were expressed as means ± standard deviations (*n* = 2). Values with different letters are significantly different (*p* < .05).

Abbreviations: N0, Wheat noodles; N3, Noodles with 3% okara; N6, Noodles with 6% okara, N9; Noodles with 9% okara; N12, Noodles with 12% okara; N15, Noodles with 15% okara.

In comparison with the control group, the hardness and adhesiveness of the N9 group increased by 107.19% and 132.14%, respectively. The reason for this result may be the influence of the abundant dietary fiber content in the okara. This efficiently encourages the starch and gluten fibers to arrange themselves in an orderly manner to generate a dense and consistent gluten–starch network structure, which enhances the hardness of the noodles (Kang et al., 2018), so the hardness of okara noodles increases with the increase of the addition amount (N0–N9). Okara can be added to noodles to efficiently increase their hardness, yet it has no negative effect on the noodles' elasticity. However, excessive insoluble dietary fiber has a negative impact on the constitution of gluten networks, which to some extent lowers the quality of the noodles (Pan et al., 2018), so as the content of okara continues to increase, noodle hardness begins to decrease (N12–N15). Therefore, it is crucial to determine the appropriate amount of okara for the quality of the noodles.

### 3.3 Sensory evaluation

**FIGURE 1:**
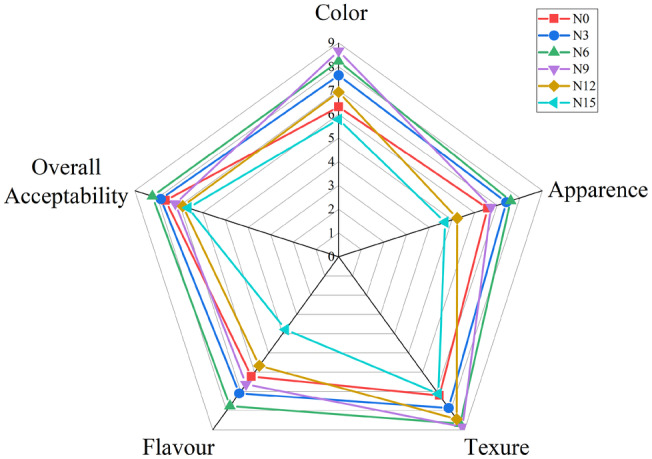
Sensory evaluation of cooked noodles with different amounts of added okara. The data are presented as mean ± SD (*n* = 20). For each parameter, a nine‐point hedonic scale ranging from 1 (dislike extremely) to 9 (like extremely) was used. N0, wheat noodles; N3, noodles with 3% okara; N6, noodles with 6% okara; N9, noodles with 9% okara; N12, noodles with 12% okara; N15, noodles with 15% okara.

The results of sensory tests were basically consistent with the changing trend of texture. As shown in Figure 1, the sensory quality of okara noodles was improved compared with the control noodles under the condition of low dosage, but the sensory quality and overall acceptability decreased with the increasing dosage. In particular, all okara noodles had a higher sensory acceptability score than 6.63 and low‐added okara noodles (N0–N6) had a higher score than 7.83. Better sensory scores for look, flavor, color, and texture are found in noodles with 6% okara content. This could be a result of the group's harder texture and brighter hue. On the contrary, the sensory score of the noodles decreased with the increase of the addition amount, which may be related to the decrease of adhesiveness and the masticatory.

### 3.4 Oxidation resistance

**FIGURE 2:**
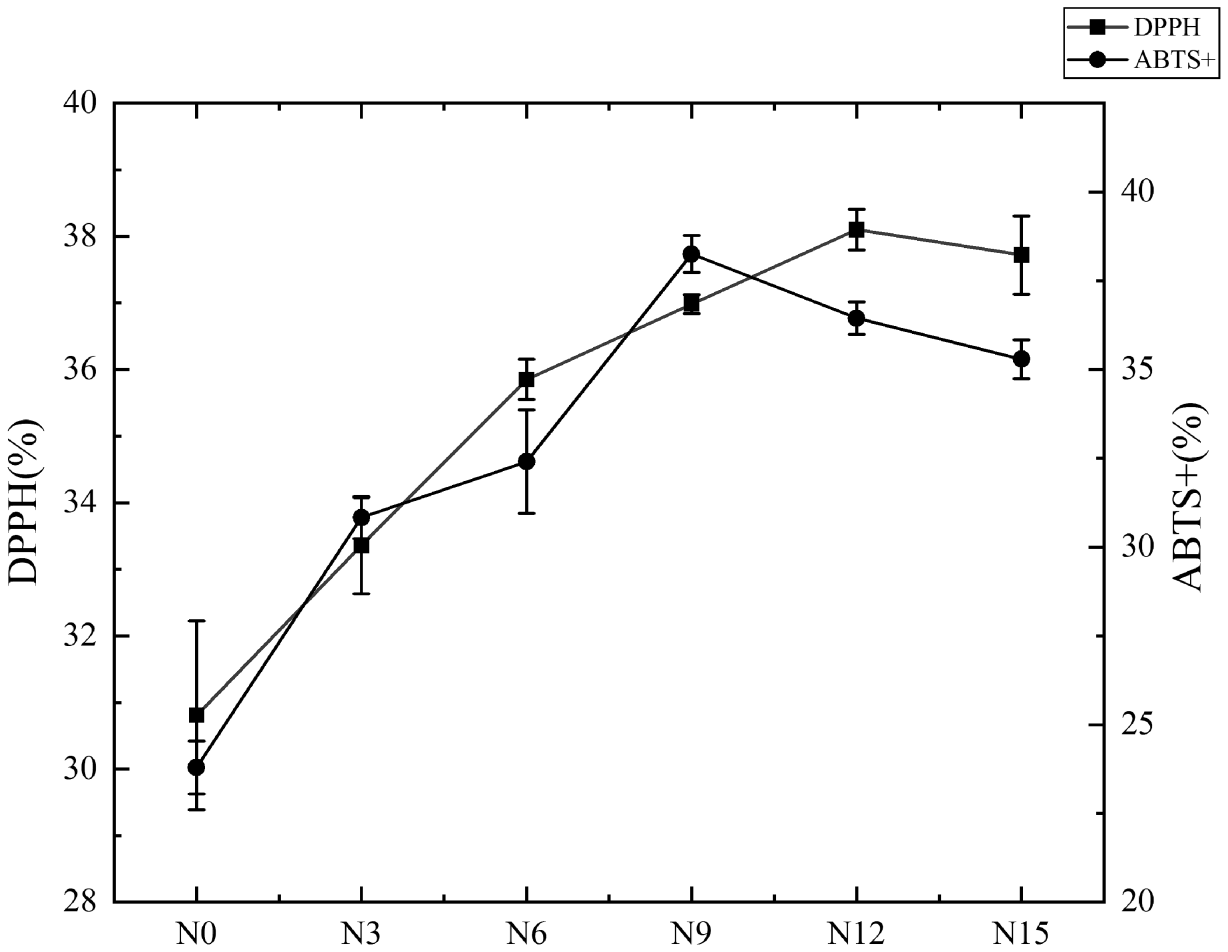
Antioxidant activity of cooked noodles with different amounts of added okara. N0, wheat noodles; N3, noodles with 3% okara; N6, noodles with 6% okara; N9, noodles with 9% okara; N12, noodles with 12% okara; N15, noodles with 15% okara.

The antioxidant properties of noodles with added okara are shown in Figure 2. From the figure, it can be observed that the DPPH radical scavenging ability and ABTS radical scavenging activity of noodles with added okara are significantly higher compared to the control group (*p* < .05). The DPPH scavenging ability and ABTS scavenging activity of okara noodles were significantly increased with the increase of okara content.

In comparison with the control group, the DPPH free radical scavenging ability of the N12 group increased by 23.66%, and the ABTS free radical scavenging activity of the N9 group increased by 60.78%. This is because okara is a sort of bioactive compound with strong prebiotic and antioxidant activities (Vital et al., 2018). Therefore, even though okara is a byproduct of processing, it can enrich the antioxidant capacity of noodle products when used as an antioxidant, combating the free radicals present in the human body (Cao et al., 2021), and providing a cost‐effective source of antioxidants for food.

### 3.5 Resistant starch content

**FIGURE 3:**
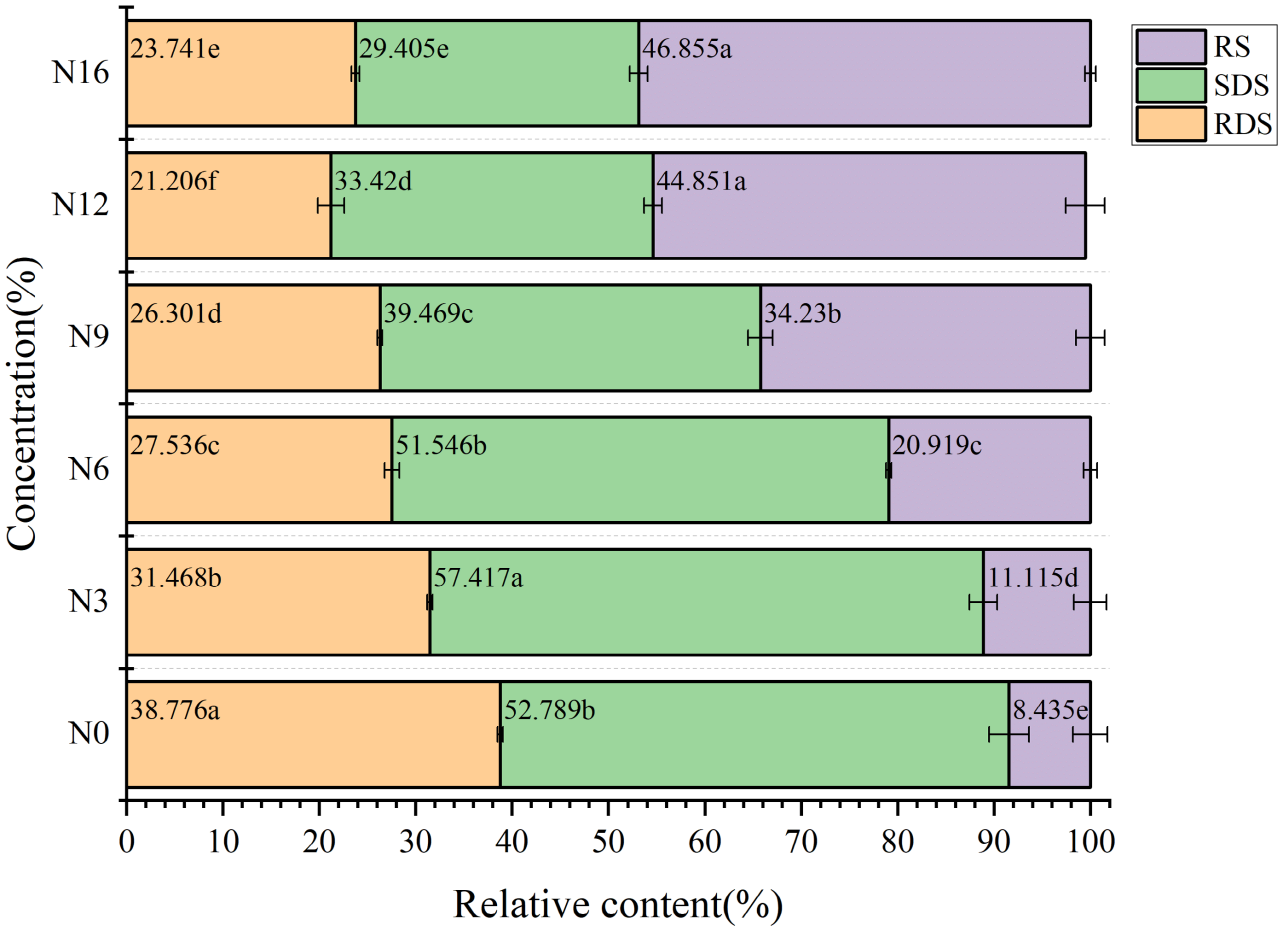
Content of SDS, RDS, and RS in cooked noodles with different amounts of added okara. The values followed by different letters were significantly different (*p* < .05). N0, wheat noodles; N3, noodles with 3% okara; N6, noodles with 6% okara; N9, noodles with 9% okara; N12, noodles with 12% okara; N15, noodles with 15% okara; RDS, rapidly digestible starch; RS, resistant starch; SDS, slowly digestible starch.

As shown in Figure 3, a complement to okara reduced the content of RDS in noodles and significantly increased the content of RS, indicating that okara reduces the digestibility of starch. Also, in okara noodles the RDS and RS values changed significantly (*p* < .05), indicating that okara reduces the digestibleness of starch. After adding okara, the RDS value decreased from 38.78% (N0) to 21.21% (N12), and the RS value increased from 8.44% (N0) to 46.86% (N15). These findings suggest that the addition of okara lessens the starch in noodles' ability to be digested. SDS has a digesting period in the colon that is five times longer than RDS among starch components and can lower postprandial blood sugar. Human digestive enzymes cannot break down RS. Microorganisms in the colon have the ability to metabolize RS, resulting in the production of physiologically useful short‐chain fatty acids and the prevention of cardiovascular illnesses (Li et al., 2022). This is because there are a large number of hydrophilic groups the dietary fiber in okara contains. Water can be held in the hydrophilic portion or the network fiber structure space when the dietary fiber's contact area and hydrophilic group grow, which can trap digestive enzymes and prevent the breakdown of starch (He et al., 2022). In addition, the gluten network structure of noodles may be affected by the protein in okara, thereby reducing the digestion rate by avoiding contact between digestive enzymes and starch particles (Xie et al., 2023). Furthermore, dietary fiber can lower the RDS level by inducing RDS to become embedded in their matrix and produce RS, which can alter how well starch is absorbed and digested (Kamble et al., 2019).

### 3.6 Amino acid content

**FIGURE 4:**
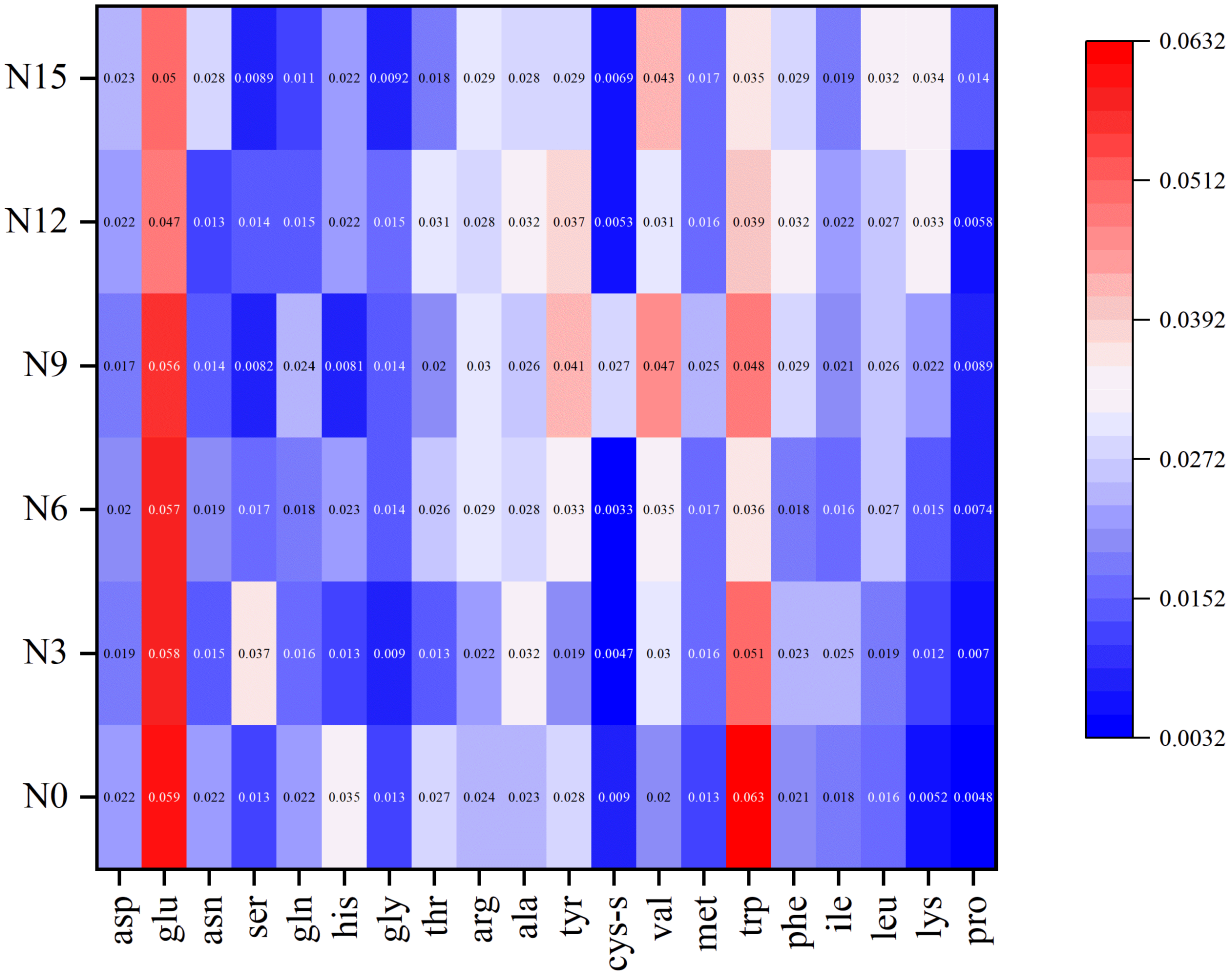
Amino acid content of cooked noodles with different amounts of added okara. N0, wheat noodles; N3, noodles with 3% okara; N6, noodles with 6% okara; N9, noodles with 9% okara; N12, noodles with 12% okara; N15, noodles with 15% okara.

The changes in the amino acid content of noodles after adding okara are shown in Figure 4. Since the first limiting amino acid in noodles is lysine, and okara contains all essential amino acids, the okara protein can produce bioactive peptides or amino acids (Vong & Liu, 2016). The data shows that in N0, the lysine content is 0.0052 mg/g, which is the first limiting amino acid. The content of lysine increased with the increase of okara addition, the lysine content in N9 increased to 0.022 mg/g, and the first limiting amino acid changed from lysine to isoleucine. Generally, increasing the substitution rate of wheat flour will increase the total amount of essential amino acids and reduce the total amount of non‐essential amino acids (Bayomy & Alamri, 2022). Therefore, to compensate for the insufficient lysine content in noodles, okara can be a good supplemental source for wheat products or a good sort of plant protein.

### 3.7 Microstructural analysis

**FIGURE 5:**
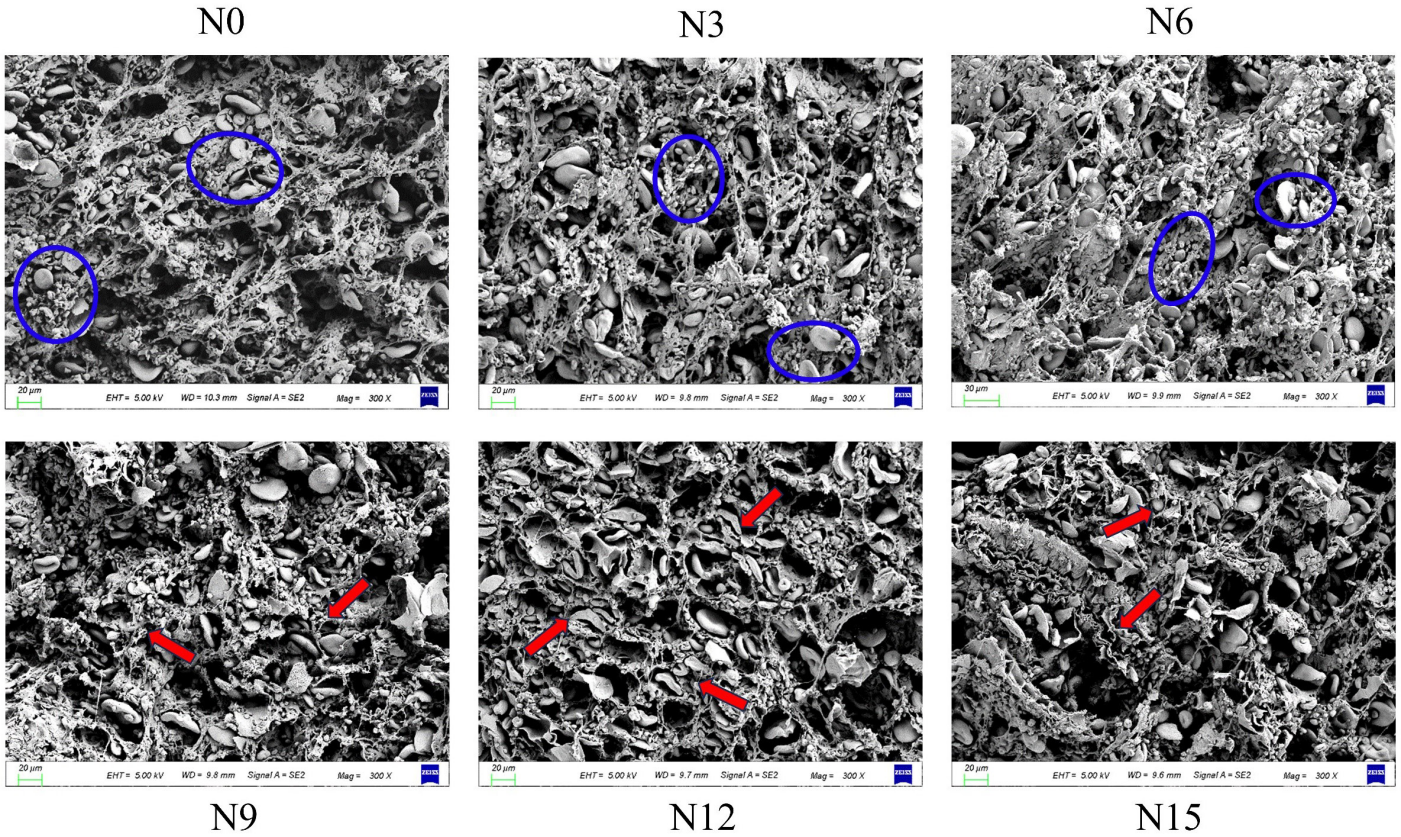
Scanning electron microscope (SEM) images of cooked noodles with different amounts of added okara. N0, wheat noodles; N3, noodles with 3% okara; N6, noodles with 6% okara; N9, noodles with 9% okara; N12, noodles with 12% okara; N15, noodles with 15% okara.

The changes in the microstructure of noodles after adding okara are shown in Figure 5. In noodles without okara (N0) and noodles with low amounts of okara (N3, N6), it can be observed that the samples exhibit a well‐developed gluten network, but the starch particles are clearly exposed on the rough surface (blue circles in the SEM image) and easily detach from the gluten network, forming a denser but looser network structure. The gluten network becomes less continuous as okara content rises, and the network structure displays a more even and well‐organized distribution, with the starch particles well encapsulated by the dense gluten network and protein matrix (red arrows in the SEM image), more firmly embedded in the reticular structure, forming a dense gluten network with a clearly strong fiber structure. This result may be the consequence of the repeated dense structure encouraging the starch and gluten fibers to cooperate in an organized manner, creating a dense and stable gluten–starch network structure that strengthens the noodles (Shang et al., 2023). Gluten proteins and starch play an important part in the protein network responsible for stability, in which dietary fiber‐rich okara interacts directly with gluten proteins, influencing the gluten–starch matrix's composition (Sun et al., 2019). Excess insoluble dietary fiber, on the other hand, has a negative impact on the structure of the gluten network, lowering the quality of the noodles (Pan et al., 2018). As a result, using a suitable amount of okara can assist in improving the quality of the noodles.

### 3.8 Pearson's correlation coefficient analysis

**FIGURE 6:**
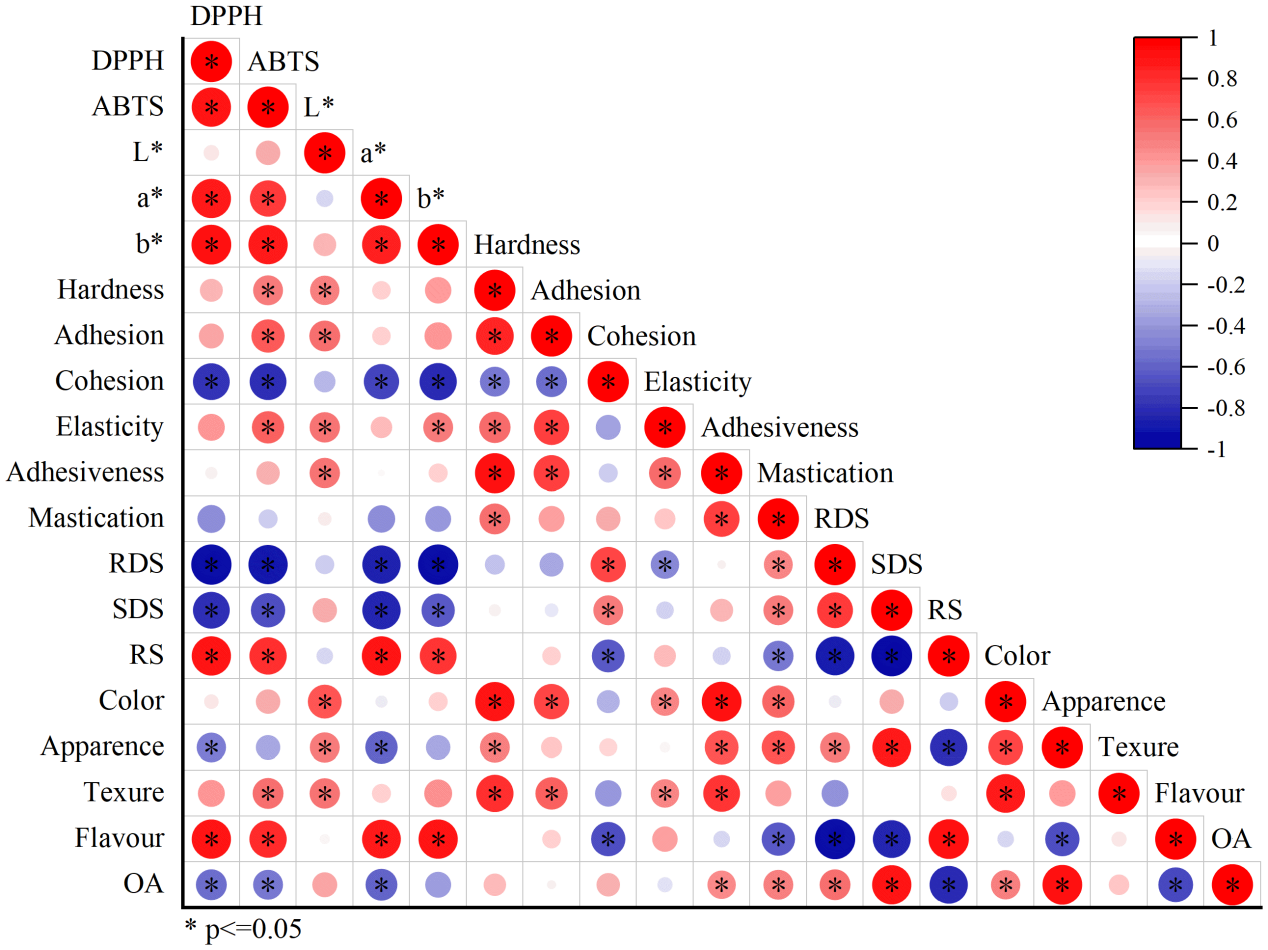
Correlation analysis of cooked noodles with different additions of okara. *Values are significantly different (*p* < .05). OA, overall acceptability; RDS, rapidly digestible starch; RS, resistant starch; SDS, slowly digestible starch.

The correlation analysis is shown in Figure 6. In order to fully understand the mechanism of the change of noodle quality characteristics caused by the addition of okara, the correlation between these parameters was analyzed. In addition to cohesion and mastication, texture parameters were positively correlated with antioxidant properties, L*, a*, and b*, RDS and SDS content, ABTS content and color were significantly positively correlated. There was a significant negative correlation between flavor and RS content. RDS and SDS were inversely correlated with antioxidant properties, a* and b*, hardness, adhesion, and elasticity. The antioxidant properties were positively correlated with L*, a*, and b*, flavor, texture parameters other than cohesion and chewability, and RS content, and negatively correlated with overall acceptability and appearance. The results showed that the addition of okara with a higher antioxidant capacity would have a larger influence on the quality of noodles. Overall acceptability is an evaluation index of consumer acceptability in sensory evaluation, indicating that although the addition of okara can improve the antioxidant capacity of noodles, it has a negative impact on consumer acceptability, which is not conducive to the practical application of products. Therefore, it is particularly critical to find the appropriate amount of okara addition.

## 4 CONCLUSION

The results showed that replacing a portion of wheat flour with okara improved the antioxidant capacity and increased the RS content of the noodles. Also, this substitution effectively inhibited starch digestion and reduced the limitation of essential amino acids to lysine. What is more, the microscopic and textural analysis also demonstrated that adding okara enhanced the strength of the noodles. These findings confirm that the usage of okara as an enhanced food or enriched product is supported academically. To completely understand the probable mechanisms by which okara produces changes in noodles, additional research should be conducted on how the dietary fiber, protein, and lipids in okara influence the quality and digestibility of fresh noodles, respectively.

## AUTHOR CONTRIBUTIONS

**Lingchen Zhou:** Conceptualization (equal); formal analysis (equal); investigation (equal); writing – original draft (equal); writing – review and editing (equal). **Sumin Gao:** Visualization (equal); writing – review and editing (equal). **Cheng Yang:** Methodology (equal). **Zhao Liu:** Writing – review and editing (equal). **Xiangren Meng:** Funding acquisition (equal); resources (equal); supervision (equal). **Zhongwen Cao:** Funding acquisition (equal); project administration (equal); resources (lead).

## CONFLICT OF INTEREST STATEMENT

The authors declare no conflicts of interest.

## Data Availability

The data that support the findings of this study are available from the corresponding author upon reasonable request.
